# Supplementary material for: Full-length transcriptomic analysis in murine and human heart reveals diversity of PGC-1α promoters and isoforms regulated distinctly in myocardial ischemia and obesity
Source: BMC Biol. 2022 Jul 30;20:169. doi: 10.1186/s12915-022-01360-w (PMC9338484; doi:10.1186/s12915-022-01360-w)
Supplement: Supplementary file 1 — Additional file 1: Figure S1: Quality Control for Primary, Secondary and Tertiary Analysis of SMRT-Sequencing. Figure S2: Sanger-Sequencing Results. Figure S3: Non-canonical exons (novel and previously known) in genomic context. Figure S4: Predicted Open reading Frame Ex1c (murine and human). Figure S5: Sanger-Sequencing Results of flanking primers approach for murine Exon1c/Exon2-junction. Table S1: Primers used for detecting starting exons with q(PCR). Figure S6: Pre-diabetic phenotype. Figure S7: Strategy for Detection of PGC1α-Isoforms in qPCR. Figure S8: Distribution of PGC-1α isoform expression in lean vs DIO under I/R. Table S2: Primers used for detecting isoform-pattern with q(PCR). Table S3: Primers used for flanking approach for Exon1c/Exon2 junction. 7 [file 12915_2022_1360_MOESM1_ESM.docx]

**
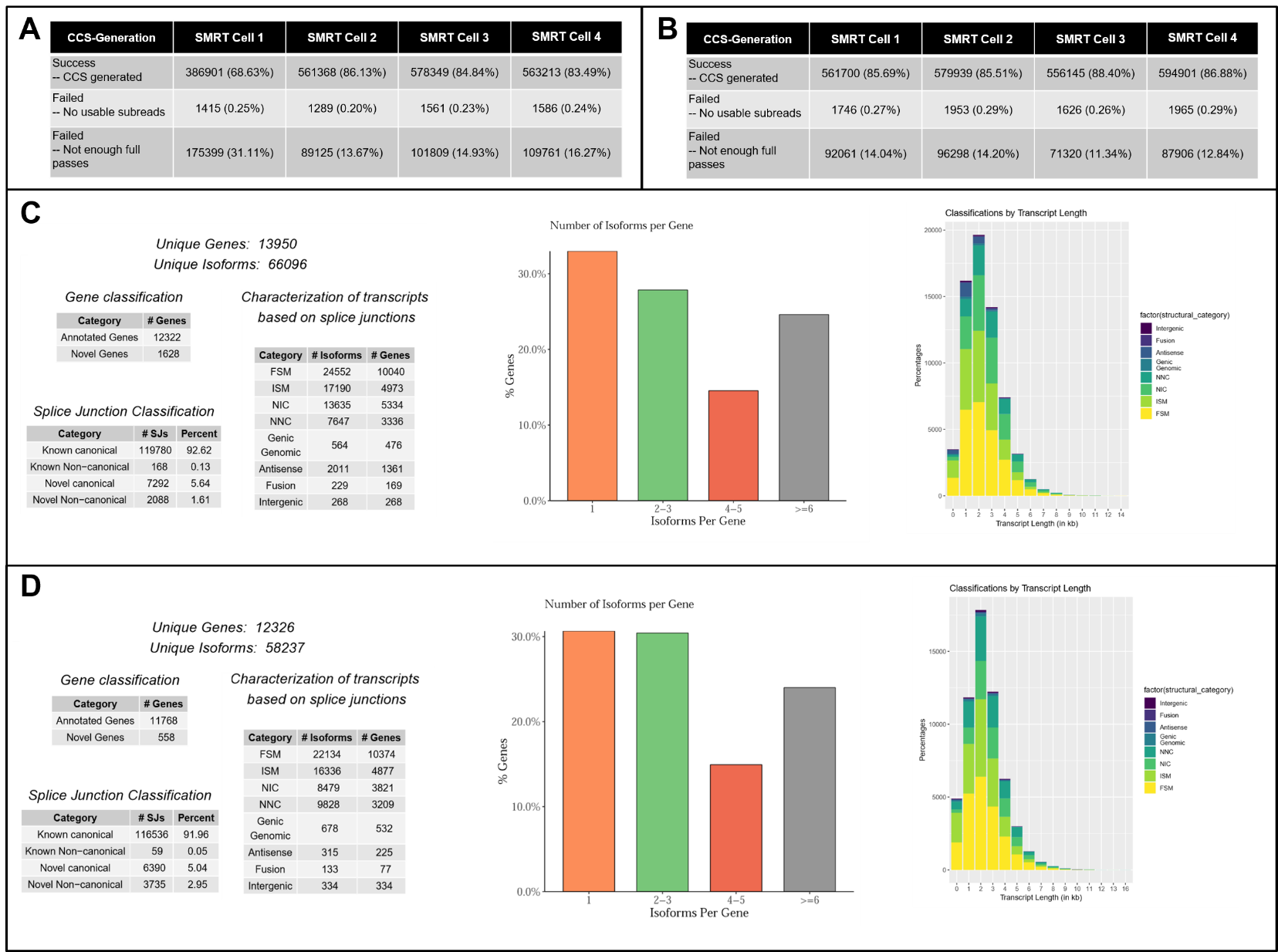
Additional Figures & Tables**

**Additional file 1: Figure S1: Quality Control for Primary, Secondary and Tertiary Analysis of SMRT-Sequencing**

Quality control outputs from different steps of analysis of human (A and C) and murine (B and D) datasets.

**A. and B.** Isoseq3-Pipeline Step 1 (Circular Consensus Reads).

**
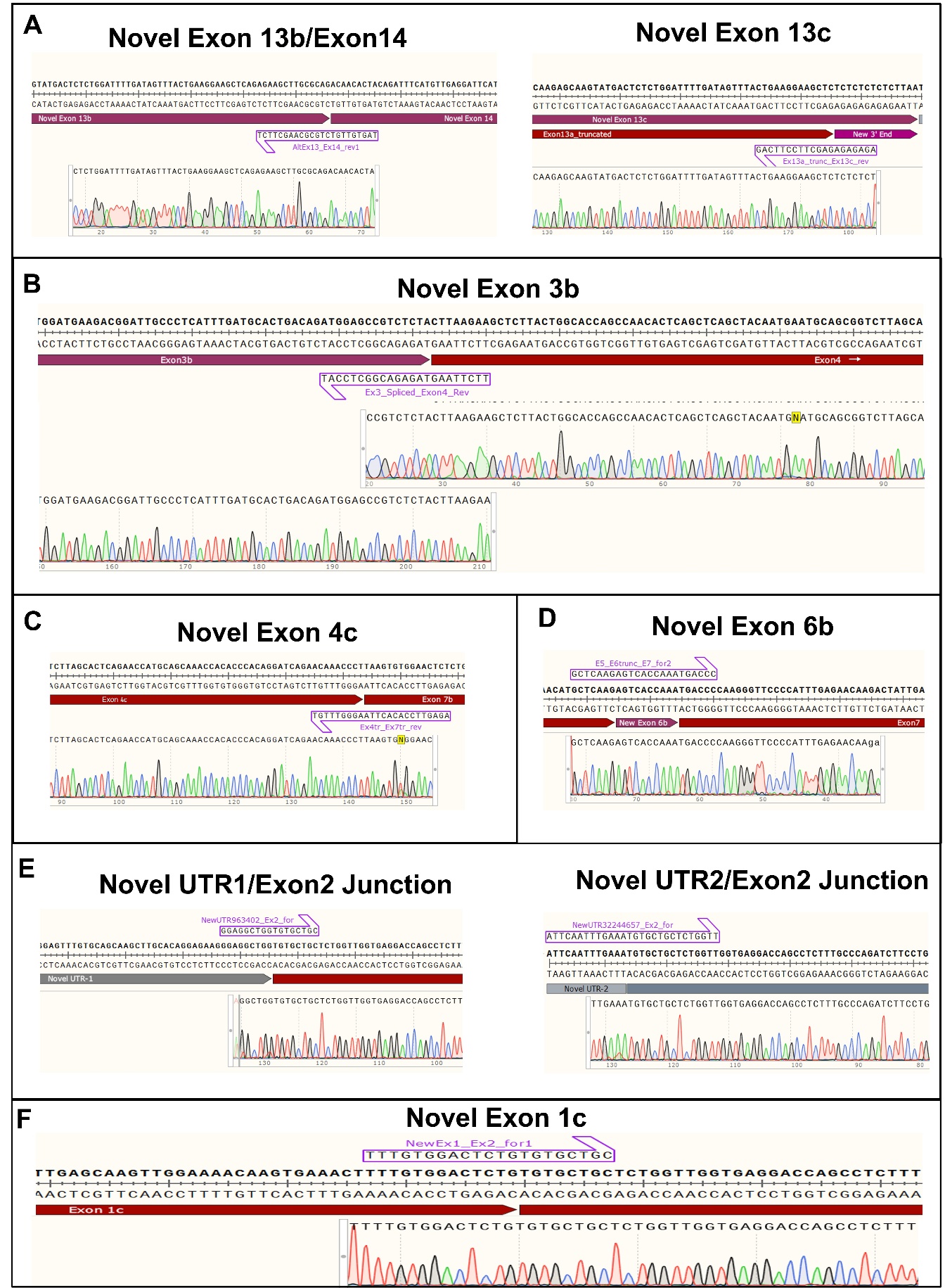
**

**Additional file 2: Figure S2: Sanger-Sequencing Results**

Shown are the exemplary results of sanger sequencing of fragments after PCR with specific primers (see Additional files 6 and 10: Tables S1 and S2) followed by gel ectrophoresis and extraction. **A.** Novel Exon 13b / Exon 14 junction (left side) as well as Novel Exon 13c (right side). **B.** Junction covering novel Exon 3b/Exon4-junction. **C.** novel Exon 4b / Exon 7b junction. **D.** Junction covering Exon5/Novel Exon 6b/Exon7-Junction. **E.** Novel UTR1-Exon2-Junction, which is unique for PGC1α-E3c (left side) and novel UTR2-Exon2-Junction, which is unique for CT-PGC1α-E3c (right side). **F.** Junction covering the novel Exon 1c and Exon 2.

**
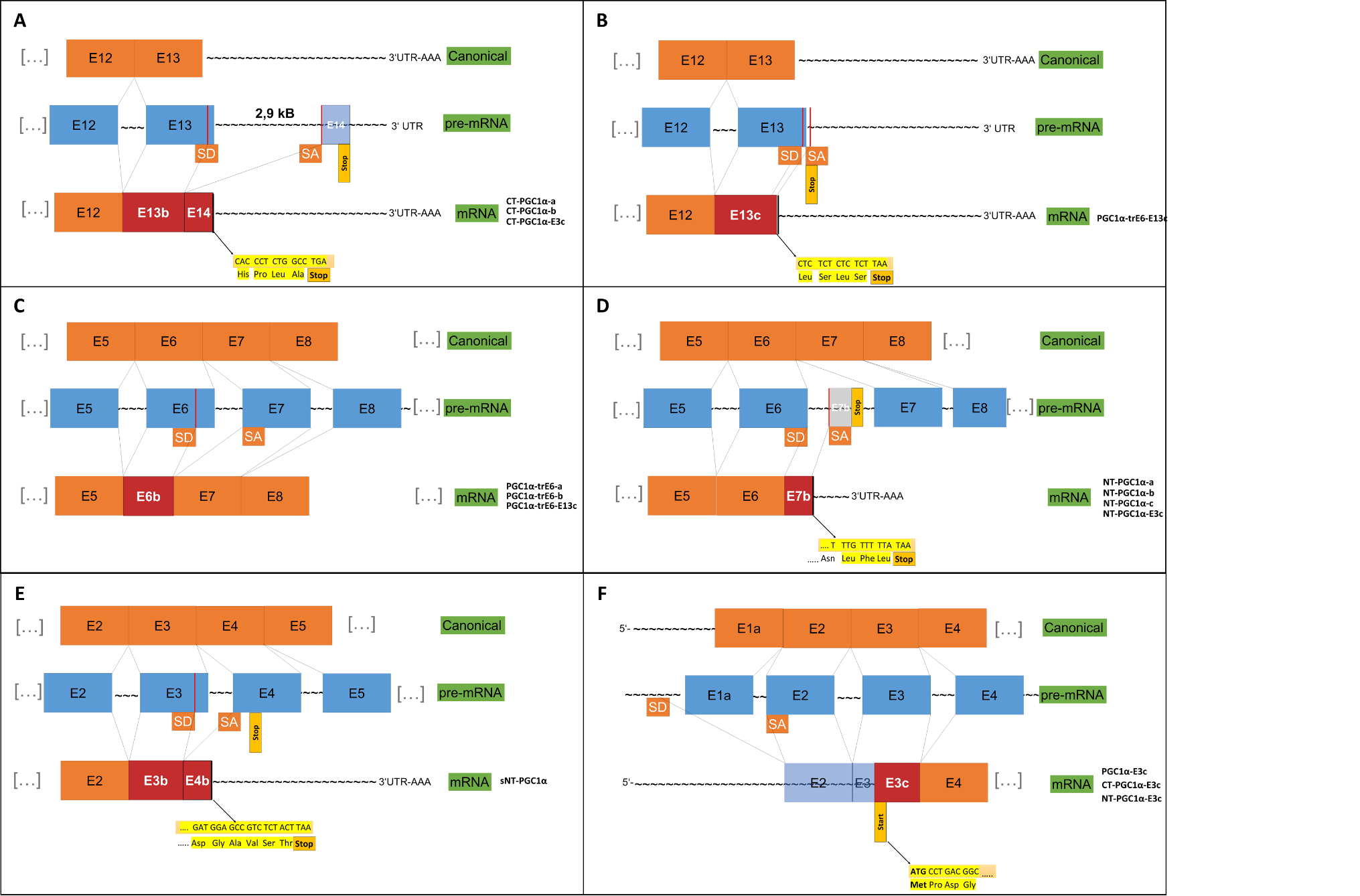
**

**Additional file 3: Figure S3: Non-canonical exons (novel and previously known) in genomic context.** Schematic overview of building mechanism of non-canonical exons used in the isoform pattern detected. Shown is the canonical sequence as reference, the pre-mRNA before splicing of introns as well as the final mRNA detected by long-read sequencing. Orange boxes show canonical and unaltered exons on mRNA level, blue boxes show exonic sequences in the genomic context / pre-mRNA, red boxes show novel resp. altered exons in relation to the canonical sequence. Boxes with transparency are used for previously intronic genomic sequences spliced into coding exons on mRNA level and therefore building novel exonic sequences. SD = splice donor site, SA = splice acceptor site, waved lines = intronic regions. Isoform names on each right side showing isoforms which include this feature (details about those isoforms in figure 3). **A.** Novel C-terminus created by splicing event in exon 13 and previous intronic / 3’UTR region, resulting in novel exons 13b and 14 including a novel stop-codon. **B.** Novel C-terminus (different from A.), created by splicing within exon 13 and resulting in novel exon 13c including novel stop codon. **C.** Novel exon 6b resulting by splicing within exon 6 with preserved open reading frame. **D.** Novel exon 7b introduced by concatenated splicing of exon 6 to region within intron 6, resulting in premature stop codon. **E.** Shortened exon 3 (named here exon 3b) spliced on exon 4 creates frameshift with novel stop codon, leading to premature stop in exon 4 (results in novel exon 4b). **F.** Alternative splicing in the canonical 5’ UTR leads to skipping of exon 1 and due to consecutive frameshift event to a novel translation start site within the canonical exon 3. The novel, shortened exon 3 as start of translation is named exon 3c.

Please note that this schematic overview is not intended to show exonic structure in scale (for true scale see figure 3).

**
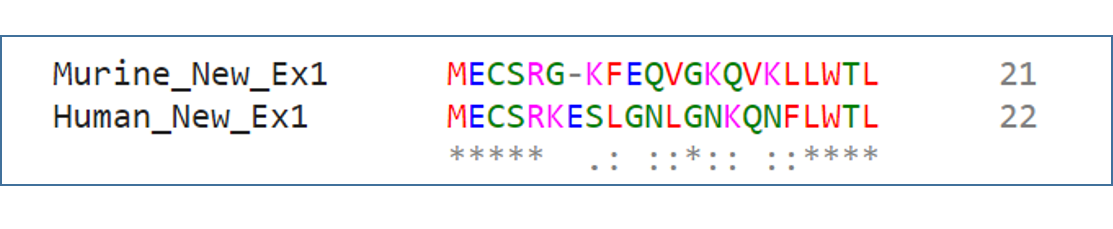
**

**Additional file 4: Figure S4: Predicted Open reading Frame Ex1c (murine and human)**

**
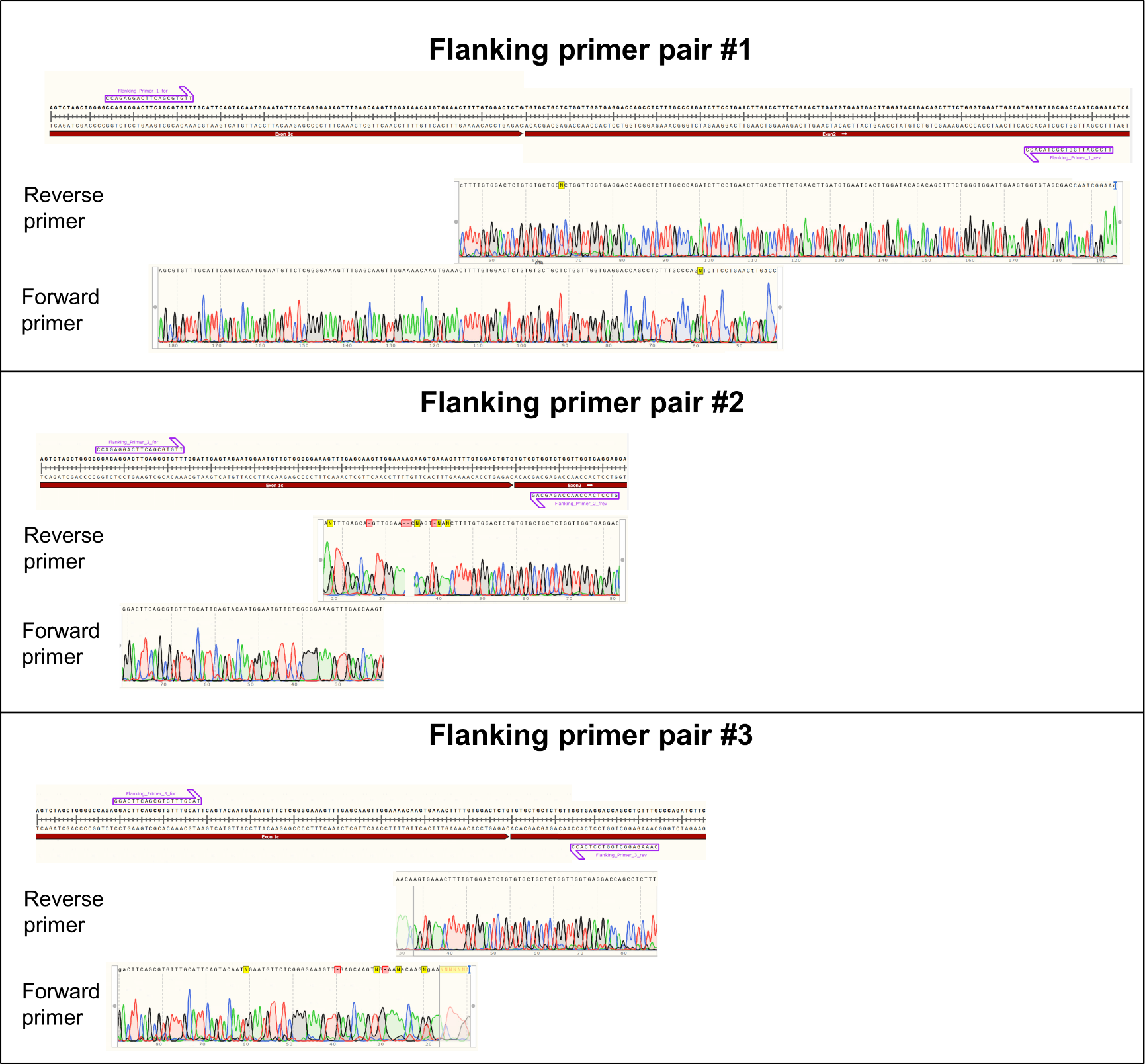
**

**A**

**B**

**C**

**Additional file 5: Figure S5: Sanger-Sequencing Results of flanking primers approach for murine Exon1c/Exon2-junction**

Shown are the results of sanger sequencing of fragments after PCR with specific primers flanking the novel Exon1c/Exon2 (see Additional file 11: table S3) followed by gel ectrophoresis and extraction. **A.** PCR Fragment generated by flanking primer pair 1. Both sequencing results using the forward and the reverse primer are shown below. **B.** PCR Fragment generated by flanking primer pair 2. Both sequencing results using the forward and the reverse primer are shown below. **C.** PCR Fragment generated by flanking primer pair 3. Both sequencing results using the forward and the reverse primer are shown below.

| **Target Name** | **Forward Primer** | **Reverse Primer** | **Junction covered** |
| --- | --- | --- | --- |
| **exon 1a** | Ex1CDS_Ex2_for:  CATAGAGTGTGCTGCTCTGGT | exon2_rev: GCTCATTGTTGTACTGGTTGGATATG | exon 1a_exon2 |
| **exon 1b** | Ex1bCDS_Ex2_for:  AATTTTGAAATGTGCTGCTCTGGT | exon2_rev: GCTCATTGTTGTACTGGTTGGATATG | exon 1b_exon2 |
| **exon 1b‘** | Ex1b'_Ex2_for:  CACTATGCTGCTGTGTGCTG | exon2_rev: GCTCATTGTTGTACTGGTTGGATATG | exon 1b‘_exon2 |
| **(novel) exon 1c** | NewEx1_Ex2_for1: TTTGTGGACTCTGTGTGCTGC | exon2_rev: GCTCATTGTTGTACTGGTTGGATATG | exon 1c_exon2 |

**Additional file 6: Table S1: Primers used for detecting starting exons with q(PCR)**

**
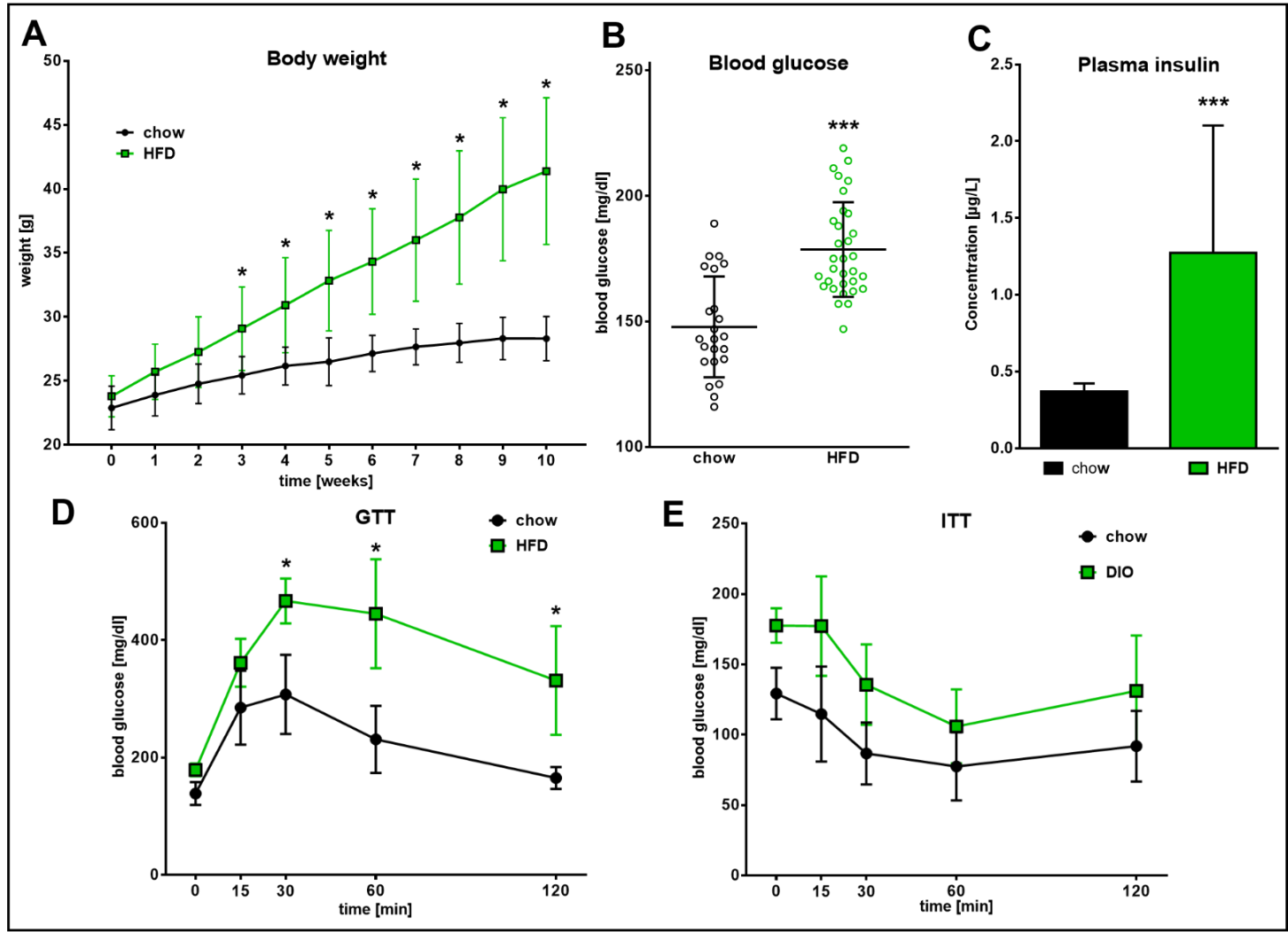
**

**Additional file 7: Figure S6: Pre-diabetic phenotype**

**A.** Summarized data for body weight development within 10 weeks of standard chow diet (SD) and High-Fat Diet (HFD), n=11. **B.** Summarized data for blood glucose of Ctrl & DIO animals after 9 weeks of feeding (n=23/32). **C.** Summarized data for plasma insulin ELISA of Ctrl & DIO animals after 10 weeks of feeding (n=15/16). **D.** Summarized data for glucose tolerance test of Ctrl & DIO animals after injecting 2 mg glucose per gram body weight after 10 weeks of feeding (n=7/8). **E.** Summarized data for insulin tolerance test of Ctrl & DIO animals after injecting 0,75 U insulin per kg body weight after 10 weeks of feeding (n=11/12).

**
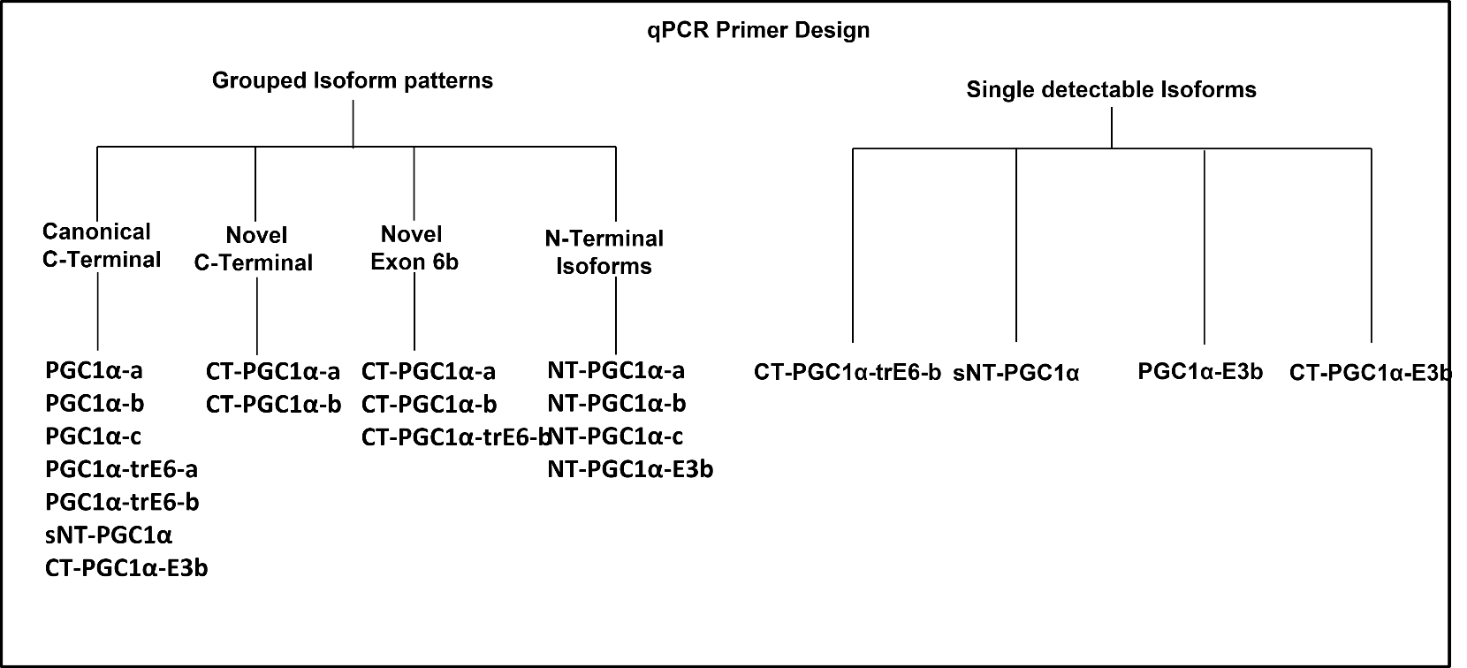
**

**Additional file 8: Figure S7: Strategy for Detection of PGC1α-Isoforms in qPCR**


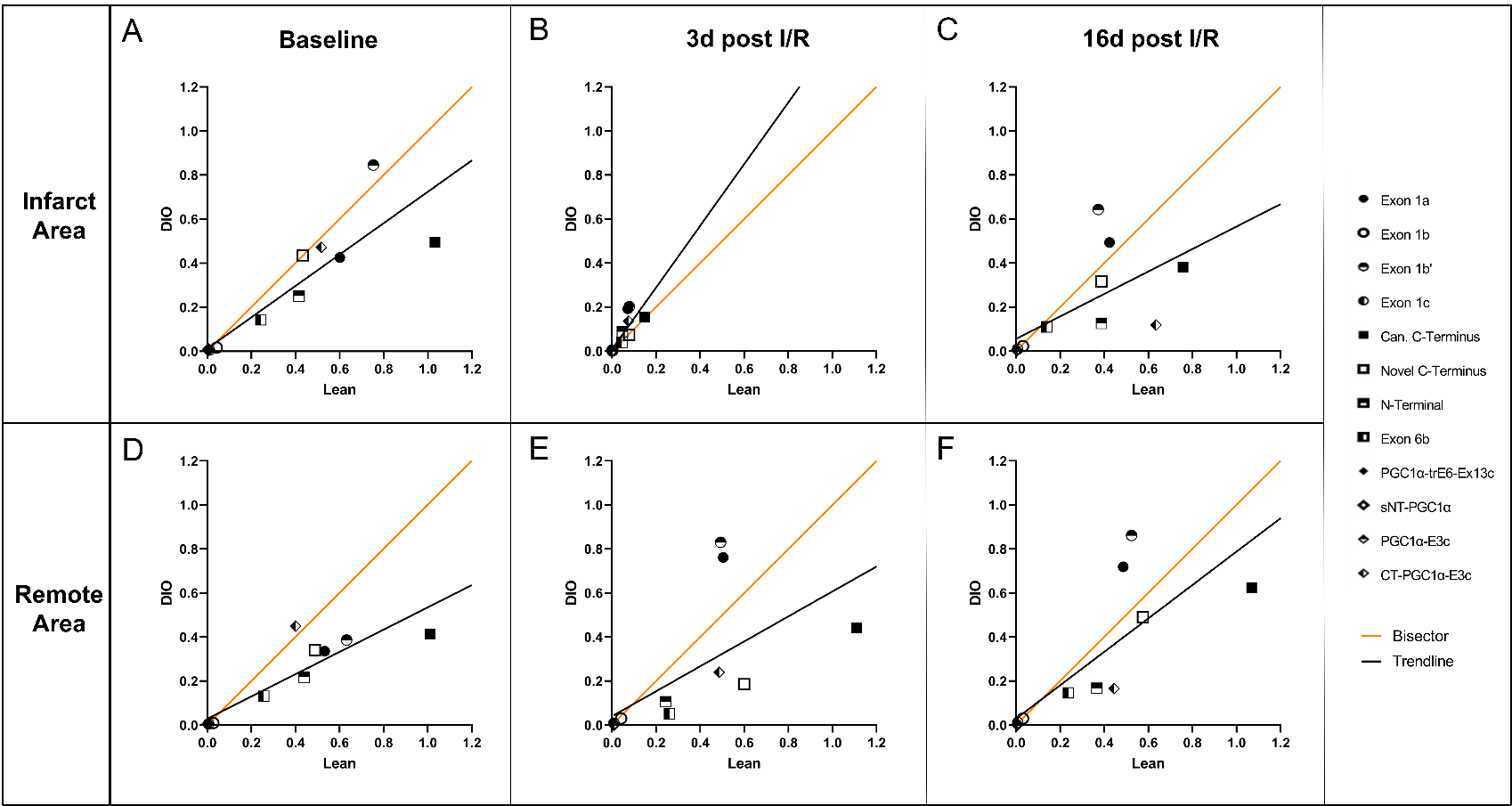


**Additional file 9: Figure S8: Distribution of PGC-1α isoform expression in lean vs DIO under I/R.** Housekeeper-normalized expression levels at baseline and 3- or 16-days post Ischemia / Reperfusion injury as relation between DIO (y-axis) or lean mice (x-axis). Shown are values for the infarcted area (upper row) and remote area (bottom row). Each dot represents average expression value (n=4) of each transcript (either promoter-wise, group-wise or single-detectable isoforms, with same categories as in Figure 5). Black line represents trendline for average expression values of all transcripts. Orange line shows bisector, where values above mean higher and underneath mean lower values in DIO than lean mice.

**A.** Baseline expression. The majority of all PGC-1α transcripts exhibit lower expression levels in DIO than lean mice, with few exceptions (see text). **B.** Expression 3 days post I/R. In contrast to baseline, in the infarcted area, all transcripts are either equally or higher expressed in DIO. **C.** Expression 16 days post I/R. In the infarcted area in contrast to remote, the combination of metabolic and ischemic hit leads to incomplete recovery of expression over time.

| **Target Name** | **Forward Primer** | **Reverse Primer** | **Covered isoforms / comments** |
| --- | --- | --- | --- |
| **Canonical C-Terminal** | Ex12_canEx13_For: TGCAGACCTAGATACCAACTCAG | canexon 13_canUTR_Rev: TGGGAACACGTTACCTGCG | PGC-1α-1, PGC-1α-b, PGC-1α-c, PGC-1α-trE6-a, PGC-1α-trE6-b, PGC-1α-trE8.2-a, sNT-PGC-1α, PGC-1α-E4c-a, CT-PGC-1α-E3c |
| **Novel C-Terminal** | AltEx13_Ex14_for1: CTCAGACGATTTTGACCCTGCT | AltEx13_Ex14_rev1 AGTGTTGTCTGCGCAAGCTTCT | CT-PGC-1α-a, CT-PGC-1α-b |
| **N-Terminal End** | Ex6_AltEx7_Rev2: AAAACAAATTTGGTGACTCTGGGG | Ex4_Ex5_junction_For: ACCCTGCCATTGTTAAGACCGA | NT-PGC-1α-a, NT-PGC-1α-b, NT-PGC-1α-c, NT-PGC-1α-E3c |
| **Novel exon 6b** | E5_E6trunc_E7_for2: GCTCAAGAGTCACCAAATGACCC | exon7_exon8_rev: GAGGAGTTAGGCCTGCAGTT | PGC-1α-trE6-a, PGC-1α-trE6-b, PGC-1α-trE6-Ex13c |
| **PGC-1α-trE6-Ex13c** | exon 12_for: TTACACCTGTGACGCTTTCG | Ex13b-Strich_Ex14b-Strich_rev: AGAGAGAGAGCTTCCTTCAG | PGC-1α-trE6-Ex13c |
| **PGC-1α-trE8.1-a** | Trunc8_trunc13UTR_for: GCTCCAAGACTCTAGACAAATCTT | Trunc8_trunc13UTR_rev1:  GATGGGCTACCCACAGTGTC | PGC-1α-trE8.1-a |
| **PGC-1α-trE8.2-a** | exon8_junctionwithin_for:  AACCAAGAGGGCCCGGTA | exon8_rev:  TTCTGGTGCTGCAAGGAGAG | Relatively unspecific binding; used only for proof of Isoform itself |
| **PGC-1α-E3d_E13c-a** | exon 1can_for_neu1: GCAGCCAAGACTCTGTATGGA | Ex3_trunc_Ex13UTR_rev: TGGGTAGAAAGTCTCCATCTGT | PGC-1α-E3d_E13c-a |
| **sNT-PGC-1α** | exon2_for: TGATGTGAATGACTTGGATACAGACA | Ex3_Spliced_exon4_Rev: TTCTTAAGTAGAGACGGCTCCAT | sNT-PGC-1α |
| **PGC-1α-E11_E13d-a** | exon8_for2:  AAGGATGCGCTCTCGTTCAA | Ex13_Ex11_Rev:  TCGTCTGAGTTGGTATCCATCAT | PGC-1α-E11_E13d-a |
| **PGC-1α-E4c-a** | exon 1can_for_neu2:  CTCAGTAAGGGGCTGGTTGC | Ex4tr_Ex7tr_rev:  GAGTTCCACACTTAAGGGTTTGT | PGC-1α-E4c-a |
| **PGC-1α-E3c** | NewUTR963402_Ex2_for:  GGAGGCTGGTGTGCTGC | exon2_rev: GCTCATTGTTGTACTGGTTGGATATG | PGC-1α-E3c |
| **CT-PGC-1α-E3c** | NewUTR32244657_Ex2_for ATTCAATTTGAAATGTGCTGCTCTGGTT | exon2_rev: GCTCATTGTTGTACTGGTTGGATATG | CT-PGC-1α-E3c |

**Additional file 10: Table S2: Primers used for detecting isoform-pattern with q(PCR)**

| **Target Name** | **Forward Primer** | **Reverse Primer** |
| --- | --- | --- |
| **Exon1c/Exon2 flanking sequence 1** | Flanking_Primer_1_for: CCAGAGGACTTCAGCGTGTT | Flanking_Primer_1_rev: TTCCGATTGGTCGCTACACC |
| **Exon1c/Exon2 flanking sequence 2** | Flanking_Primer_2_for: CCAGAGGACTTCAGCGTGTT | Flanking_Primer_2_rev: GTCCTCACCAACCAGAGCAG |
| **Exon1c/Exon2 flanking sequence 3** | Flanking_Primer_3_for:  CAAAGAGGCTGGTCCTCACC | Flanking_Primer_3_rev:  GGACTTCAGCGTGTTTGCAT |

**Additional file 11: Table S3: Primers used for flanking approach for Exon1c/Exon2 junction**
